# 3-(1*H*-Imidazol-1-yl)propanaminium picrate

**DOI:** 10.1107/S1600536813025646

**Published:** 2013-09-21

**Authors:** T. S. Yamuna, Jerry P. Jasinski, Courtney E. Duff, H. S. Yathirajan, Manpreet Kaur

**Affiliations:** a Department of Studies in Chemistry, University of Mysore, Manasagangotri, Mysore 570 006, India; b Department of Chemistry, Keene State College, 229 Main Street, Keene, NH 03435-2001, USA

## Abstract

In the title salt [systematic name: 3-(1*H*-imidazol-1-yl)propanaminium 2,4,6-tri­nitro­phenolate], C_6_H_12_N_3_^+^·C_6_H_2_N_3_O_7_^−^, there are five independent cation–anion pairs (*A*, *B*, *C*, *D*, *E*) in the asymmetric unit. In the cation, the ammonium group is protonated with the amino­propyl group nearly at right angles to the mean plane of the imidazole ring showing C—N—C—C torsion angles ranging from 79.6 (2) to 99.79 (19)° in the five cations. The nitro groups in the anion are twisted from the benzene mean plane with maximum dihedral angles subtended by nitro substituents *ortho* to the phenolate O atom of 26.0 (2) and 37.3 (7) (*A*), 28.9 (5) and 35.3 (1) (*B*), 34.7 (7) and 36.9 (7) (*C*), 14.7 (4) and 36.9 (2) (*D*) and 33.1 (1) and 35.4 (3)° (*E*). In contrast, the nitro groups in the *para* positions lie much closer to the aromatic ring plane, subtending dihedral angles of 1.8 (3) (*A*), 3.5 (3) (*B*), 6.03 (*C*), 2.1 (3) (*D*) and 7.7 (1)° (*E*). Disorder is observed for one O atom of an *ortho* nitro group in anion *D* with an occupancy ratio of 0.53 (5):0.47 (5). In the crystal, N—H⋯O cation–anion and N—H⋯N cation–cation hydrogen bonds are observed, linking the ions into chains along [010]. In addition, weak C—H⋯O cation–anion inter­actions occur.

## Related literature

For pharmacological properties of imidazole compounds, see: Lombardino & Wiseman (1974 ▶). For applications of substituted imidazoles, see: Maier *et al.* (1989*a* ▶,*b* ▶). For imidazole derivatives as anti­cancer agents, see: Krezel (1998 ▶). For electrostatic or hydrogen-bonding inter­actions in picric acid charge-transfer complexes, see: In *et al.* (1997 ▶). For imidazolium-based cation picrate salts as good candidates for energetic ionic salts, see: Jin *et al.* (2005 ▶). For the crystal structure of imidazolium picrate, see: Soriano-García *et al.* (1990 ▶) and for the structures of picrates of some other imidazole deriv­atives, see: Du & Zhao (2003 ▶); Dutkiewicz *et al.* (2011 ▶); MacDonald *et al.* (2005 ▶); Nardelli *et al.* (1987 ▶); Pi *et al.* (2009 ▶). For standard bond lengths, see: Allen *et al.* (1987 ▶).
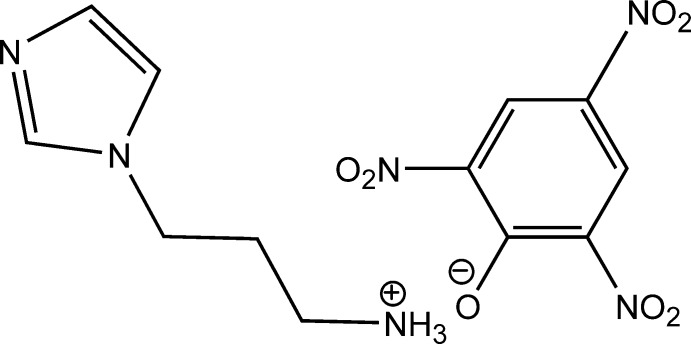


## Experimental

### 

#### Crystal data

C_6_H_12_N_3_^+^·C_6_H_2_N_3_O_7_^−^
*M* *_r_* = 354.29
Monoclinic, 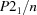

*a* = 11.98275 (18) Å
*b* = 38.5234 (6) Å
*c* = 16.4239 (2) Å
β = 94.1970 (14)°
*V* = 7561.2 (2) Å^3^
*Z* = 20
Cu *K*α radiation
μ = 1.13 mm^−1^
*T* = 173 K
0.21 × 0.17 × 0.08 mm

#### Data collection

Agilent Xcalibur (Eos, Gemini) diffractometer
Absorption correction: multi-scan (*CrysAlis PRO* and *CrysAlis RED*; Agilent, 2012 ▶) *T*_min_ = 0.870, *T*_max_ = 1.000
52087 measured reflections
14795 independent reflections
12167 reflections with *I* > 2σ(*I*)
*R*_int_ = 0.032

#### Refinement

*R*[*F* ^2^ > 2σ(*F* ^2^)] = 0.042
*wR*(*F* ^2^) = 0.116
*S* = 1.02
14795 reflections
1197 parameters
12 restraints
H atoms treated by a mixture of independent and constrained refinement
Δρ_max_ = 0.51 e Å^−3^
Δρ_min_ = −0.29 e Å^−3^

### 

Data collection: *CrysAlis PRO* (Agilent, 2012 ▶); cell refinement: *CrysAlis PRO*; data reduction: *CrysAlis RED* (Agilent, 2012 ▶); program(s) used to solve structure: *SUPERFLIP* (Palatinus & Chapuis, 2007 ▶); program(s) used to refine structure: *SHELXL2012* (Sheldrick, 2008 ▶); molecular graphics: *OLEX2* (Dolomanov *et al.*, 2009 ▶); software used to prepare material for publication: *OLEX2*.

## Supplementary Material

Crystal structure: contains datablock(s) I. DOI: 10.1107/S1600536813025646/sj5352sup1.cif

Structure factors: contains datablock(s) I. DOI: 10.1107/S1600536813025646/sj5352Isup2.hkl

Supplementary material file. DOI: 10.1107/S1600536813025646/sj5352Isup3.cml

Additional supplementary materials:  crystallographic information; 3D view; checkCIF report

## Figures and Tables

**Table 1:** Hydrogen-bond geometry (Å, °)

*D*—H⋯*A*	*D*—H	H⋯*A*	*D*⋯*A*	*D*—H⋯*A*
N6*A*—H6*AA*⋯O3*E* ^i^	0.85 (2)	2.06 (2)	2.8943 (18)	167.5 (18)
N6*A*—H6*AB*⋯O3*D* ^ii^	0.91 (2)	2.08 (2)	2.8671 (18)	143.1 (16)
N6*A*—H6*AB*⋯O4*D* ^ii^	0.91 (2)	2.25 (2)	2.961 (2)	134.6 (15)
N6*A*—H6*AC*⋯N4*C*	0.95 (2)	1.87 (2)	2.8157 (19)	172.5 (18)
N6*B*—H6*BA*⋯O3*B* ^iii^	0.92 (2)	2.117 (19)	2.8728 (17)	138.5 (15)
N6*B*—H6*BA*⋯O4*B* ^iii^	0.92 (2)	2.268 (19)	3.006 (2)	136.6 (15)
N6*B*—H6*BB*⋯O3*B*	0.89 (2)	2.00 (2)	2.8502 (17)	161.0 (19)
N6*B*—H6*BC*⋯N4*A* ^iv^	0.92 (2)	1.88 (2)	2.7988 (19)	173.4 (17)
N6*C*—H6*CA*⋯O2*C* ^v^	0.85 (2)	2.305 (18)	2.8334 (19)	120.9 (15)
N6*C*—H6*CA*⋯O3*C* ^v^	0.85 (2)	2.10 (2)	2.8944 (18)	155.4 (17)
N6*C*—H6*CB*⋯O3*A* ^vi^	0.89 (2)	2.174 (19)	2.9145 (18)	140.2 (16)
N6*C*—H6*CB*⋯O4*A* ^vi^	0.89 (2)	2.270 (19)	2.986 (2)	137.3 (15)
N6*C*—H6*CC*⋯N4*D* ^vi^	0.92 (2)	1.91 (2)	2.8179 (19)	167.6 (17)
N6*D*—H6*DA*⋯O2*A* ^vii^	0.89 (2)	2.361 (19)	2.9631 (19)	124.9 (15)
N6*D*—H6*DA*⋯O3*A* ^vii^	0.89 (2)	2.06 (2)	2.8340 (17)	145.0 (16)
N6*D*—H6*DB*⋯O3*C* ^vi^	0.89 (2)	2.16 (2)	2.9171 (17)	142.0 (17)
N6*D*—H6*DB*⋯O4*C* ^vi^	0.89 (2)	2.27 (2)	2.9323 (19)	130.5 (16)
N6*D*—H6*DC*⋯N4*E*	0.89 (2)	1.91 (2)	2.7932 (19)	174.0 (17)
N6*E*—H6*EA*⋯O2*D* ^viii^	0.91 (2)	2.32 (2)	2.973 (2)	128.7 (17)
N6*E*—H6*EA*⋯O3*D* ^viii^	0.91 (2)	2.06 (2)	2.8527 (18)	145.4 (18)
N6*E*—H6*EB*⋯O3*E*	0.87 (2)	2.19 (2)	2.9056 (18)	139.6 (17)
N6*E*—H6*EB*⋯O4*E*	0.87 (2)	2.33 (2)	3.010 (2)	135.3 (16)
N6*E*—H6*EC*⋯N4*B* ^viii^	0.93 (2)	1.85 (2)	2.7750 (19)	174.0 (19)
C8*A*—H8*A*⋯O2*B* ^ix^	0.95	2.46	3.0887 (19)	123
C9*A*—H9*A*⋯O5*E*	0.95	2.43	3.243 (2)	144
C12*A*—H12*B*⋯O7*A* ^ix^	0.99	2.46	3.313 (2)	145
C9*B*—H9*B*⋯O5*B* ^i^	0.95	2.37	3.224 (2)	150
C12*B*—H12*D*⋯O7*C* ^vi^	0.99	2.47	3.335 (2)	145
C8*C*—H8*C*⋯O5*C* ^vi^	0.95	2.33	3.194 (2)	152
C9*C*—H9*C*⋯O2*E* ^i^	0.95	2.49	3.105 (2)	123
C7*D*—H7*D*⋯O5*A* ^vi^	0.95	2.35	3.233 (2)	155
C11*D*—H11*H*⋯O6*A* ^vi^	0.99	2.55	3.421 (2)	147
C12*D*—H12*G*⋯O6*B* ^vii^	0.99	2.43	3.238 (2)	139
C9*E*—H9*E*⋯O5*D* ^ii^	0.95	2.27	3.162 (6)	156
C9*E*—H9*E*⋯O5*DA* ^ii^	0.95	2.56	3.357 (17)	141
C12*E*—H12*I*⋯O6*E* ^viii^	0.99	2.34	3.186 (2)	142

Symmetry codes: (i) 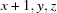; (ii) 
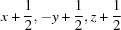
; (iii) 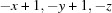; (iv) 
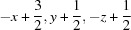
; (v) 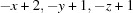; (vi) 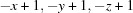; (vii) 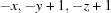; (viii) 
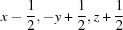
; (ix) 
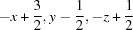
.

**Fig. 1.**
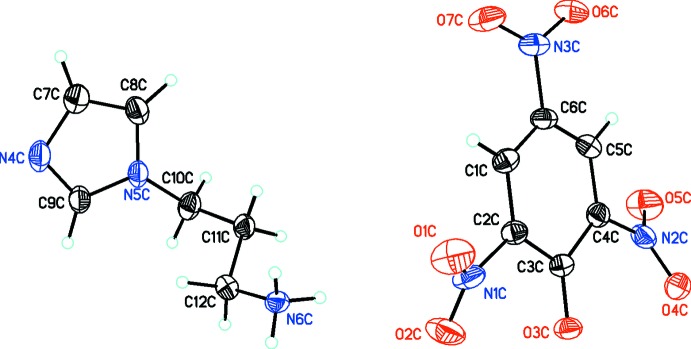
ORTEP drawing of one of five independent cation (C6H12N3+ ) and anion (C6H2N3O7-) pairs (C) in the asymmetric unit of (I), for clarity, showing the atom labeling scheme and 30% probability displacement ellipsoids.

**Fig. 2.**
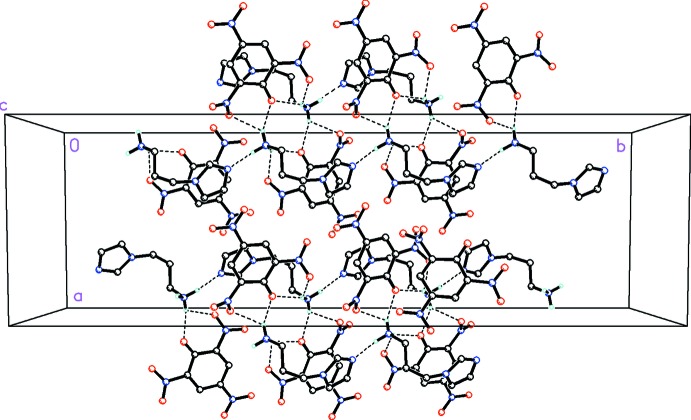
Molecular packing for (I) viewed along the c axis. Dashed lines indicate N—H···O cation–anion and N—H···N cation-cation hydrogen bonds, dahed lines, linking the ions into chains along [0 1 0]. Hydrogen atoms not involved in hydrogen bonding are omitted for clarity.

## supplementary crystallographic information

### 1. Comment

Compounds with an imidazole ring system have many pharmacological properties and play important roles in biochemical processes (Lombardino & Wiseman, 1974). Many imidazole derivatives are characterized as inhibitors of fungicides and herbicides, plant growth regulators and therapeutic agents (Maier *et al.*, 1989*a*,*b*). Imidazole derivatives are also used as potential anticancer agents (Krezel, 1998) and display a broad spectrum of pharmacological activities. It is well known that picric acid forms charge transfer molecular complexes with a number of aromatic compounds such as aromatic amines through electrostatic or hydrogen bonding interactions (In *et al.*, 1997). Picric acid is a polynitrogen compound with explosive character and imidazolium-based cation picrate salts are good candidates for energetic ionic salts (Jin *et al.*, 2005).

The crystal structures of some imidazolium picrates have been reported, for instance imidazolium picrate itself (Soriano-García *et al.*, 1990), also two solvates (hydrate and ethanolate) of 2-aminohistamine dipicrate (Nardelli *et al.*, 1987), 4-hydroxy methylimidazolium picrate (Du & Zhao, 2003), two polymorphs of betaine bis(diimidazolium) dipicrate (MacDonald *et al.*, 2005) and 3-benzyl-1-methyl-imidazolium picrate (Pi *et al.*, 2009). Recently, we have reported the crystal structure of 2-methylimidazolium picrate (Dutkiewicz *et al.*, 2011).

In view of the importance of imidazoles, this paper reports the crystal structure of the title salt, C_6_H_12_N_3_^+^ . C_6_H_2_N_3_O_7_^-^, (I).

In (I), there are five cation-anion independent pairs (A, B, C, D, E) in the asymmetric unit. For clarity only the C cation-anion pair is displayed in Fig. 1. In the cation the ammonium group is protonated with the aminopropyl group nearly at right angles to the imidazole ring showing C/N/C/C torsion angles ranging from 79.6 (2)° to 99.79 (19)° in the five cations. Bond lengths are in normal ranges (Allen *et al.*, 1987). The nitro groups in the anion are twisted from the benzene mean plane with maximum dihedral angles subtended by nitro substituents ortho to phenolate oxygen of 26.0 (2)°, 37,3(7)° (A); 28,9(5)°, 35.3 (1)° (B); 34.7 (7)°, 36.9 (7)° (C); 14.7 (4)°, 36.9 (2)° (D); 33.1 (1)°, 35.4 (3)° (E). In contrast the nitro groups in the para positions lie much closer to the aromatic ring plane with angles 1.8 (3)° (A); 3.5 (3)° (B); 6.03° (C); 2.1 (3)° (D); 7.7 (1)° (E), respectively. Disorder is observed in atom O5D, an ortho nitro group in anion D, with an occupancy ratio of 0.53 (5):0.47 (5). In the crystal, N—H···O cation-anion and N—H···N cation-cation hydrogen bonds (Table 1) are observed linking the ions into chains along [0 1 0] (Fig. 2). In addition, weak C—H···O cation-anion intermolecular interactions also contribute to packing stability.

### 2. Experimental

Commercially available 1-(3-aminopropyl)imidazole (0.5 g, 3.99 mmol) and picric acid (1.19 g, 3.99 mmol) were dissolved in 10 ml of ethanol and stirred for 5 minutes at room temperature. After 30 mins, crystals were formed on evaporation of ethanol (m.p.: 370–373 K).

### 3. Refinement

H6AA, H6AB, H6AC, H6BA, H6BB, H6BC, H6CA, H6CB, H6CC, H6DA, H6DB, H6DC, H6EA, H6EB, H6EC, were located by a difference map and refined isotropically. All of the remaining H atoms were placed in their calculated positions and then refined using the riding model with Atom—H lengths of 0.95Å (aromatic) or 0.99Å (CH_2_). Isotropic displacement parameters for these atoms were set to 1.2 (CH, CH_2_) times *U*_eq_ of the parent atom. Disorder is observed in atom O5D of anion D with an occupancy ratio of 0.53 (5):0.47 (5).

### Crystal data

**Table:**

C_6_H_12_N_3_^+^·C_6_H_2_N_3_O_7_^−^	*F*(000) = 3680
*M**_r_* = 354.29	*D*_x_ = 1.556 Mg m^−^^3^
Monoclinic, *P*2_1_/*n*	Cu *K*α radiation, λ = 1.54184 Å
*a* = 11.98275 (18) Å	Cell parameters from 17325 reflections
*b* = 38.5234 (6) Å	θ = 3.4–72.3°
*c* = 16.4239 (2) Å	µ = 1.13 mm^−^^1^
β = 94.1970 (14)°	*T* = 173 K
*V* = 7561.2 (2) Å^3^	Irregular, clear yellow
*Z* = 20	0.21 × 0.17 × 0.08 mm

### Data collection

**Table:**

Agilent Xcalibur (Eos, Gemini) diffractometer	14795 independent reflections
Radiation source: Enhance (Cu) X-ray Source	12167 reflections with *I* > 2σ(*I*)
Detector resolution: 16.0416 pixels mm^-1^	*R*_int_ = 0.032
ω scans	θ_max_ = 72.5°, θ_min_ = 3.5°
Absorption correction: multi-scan (*CrysAlis PRO* and *CrysAlis RED*; Agilent, 2012)	*h* = −9→14
*T*_min_ = 0.870, *T*_max_ = 1.000	*k* = −47→46
52087 measured reflections	*l* = −20→20

### Refinement

**Table:**

Refinement on *F*^2^	Hydrogen site location: mixed
Least-squares matrix: full	H atoms treated by a mixture of independent and constrained refinement
*R*[*F*^2^ > 2σ(*F*^2^)] = 0.042	*w* = 1/[σ^2^(*F*_o_^2^) + (0.0585*P*)^2^ + 2.3598*P*] where *P* = (*F*_o_^2^ + 2*F*_c_^2^)/3
*wR*(*F*^2^) = 0.116	(Δ/σ)_max_ = 0.001
*S* = 1.02	Δρ_max_ = 0.51 e Å^−^^3^
14795 reflections	Δρ_min_ = −0.29 e Å^−^^3^
1197 parameters	Extinction correction: *SHELXL*, Fc^*^=kFc[1+0.001xFc^2^λ^3^/sin(2θ)]^-1/4^
12 restraints	Extinction coefficient: 0.000142 (14)

### Special details

**Table:**

Geometry. All esds (except the esd in the dihedral angle between two l.s. planes) are estimated using the full covariance matrix. The cell esds are taken into account individually in the estimation of esds in distances, angles and torsion angles; correlations between esds in cell parameters are only used when they are defined by crystal symmetry. An approximate (isotropic) treatment of cell esds is used for estimating esds involving l.s. planes.

### Fractional atomic coordinates and isotropic or equivalent isotropic displacement parameters (Å^2^)

**Table:**

	*x*	*y*	*z*	*U*_iso_*/*U*_eq_	Occ. (<1)
O1A	0.06660 (12)	0.70857 (4)	0.28122 (10)	0.0543 (4)	
O2A	0.03780 (11)	0.68796 (4)	0.40019 (10)	0.0477 (4)	
O3A	0.11234 (9)	0.62090 (3)	0.40908 (7)	0.0296 (3)	
O4A	0.21360 (12)	0.56043 (3)	0.37863 (10)	0.0491 (4)	
O5A	0.39119 (12)	0.56511 (4)	0.39119 (12)	0.0636 (5)	
O6A	0.55513 (10)	0.65993 (4)	0.25325 (8)	0.0417 (3)	
O7A	0.46158 (11)	0.70758 (4)	0.23205 (9)	0.0446 (3)	
N1A	0.09133 (12)	0.68950 (4)	0.33963 (10)	0.0329 (3)	
N2A	0.29916 (12)	0.57781 (4)	0.37670 (9)	0.0321 (3)	
N3A	0.47021 (12)	0.67773 (4)	0.25701 (9)	0.0318 (3)	
C1A	0.27832 (13)	0.68191 (4)	0.29857 (10)	0.0259 (3)	
H1A	0.2729	0.7046	0.2761	0.031*	
C2A	0.19050 (12)	0.66773 (4)	0.33624 (10)	0.0245 (3)	
C3A	0.18956 (12)	0.63312 (4)	0.37119 (9)	0.0224 (3)	
C4A	0.29093 (13)	0.61429 (4)	0.35572 (9)	0.0246 (3)	
C5A	0.38185 (13)	0.62869 (4)	0.32185 (9)	0.0260 (3)	
H5A	0.4482	0.6155	0.3178	0.031*	
C6A	0.37572 (13)	0.66236 (4)	0.29393 (9)	0.0260 (3)	
O1B	0.41038 (10)	0.62930 (3)	0.10159 (8)	0.0377 (3)	
O2B	0.50153 (9)	0.58259 (3)	0.13563 (9)	0.0385 (3)	
O3B	0.37850 (9)	0.52841 (3)	0.06265 (7)	0.0261 (2)	
O4B	0.25989 (12)	0.47144 (3)	0.10774 (9)	0.0432 (3)	
O5B	0.08374 (12)	0.48134 (4)	0.08146 (10)	0.0571 (4)	
O6B	−0.05515 (10)	0.58211 (4)	0.20725 (8)	0.0392 (3)	
O7B	0.04050 (10)	0.62992 (3)	0.20991 (8)	0.0386 (3)	
N1B	0.02960 (11)	0.59876 (4)	0.19519 (8)	0.0299 (3)	
N2B	0.17976 (12)	0.49122 (4)	0.10008 (9)	0.0331 (3)	
N3B	0.41401 (11)	0.59841 (3)	0.11880 (8)	0.0243 (3)	
C1B	0.22192 (12)	0.59790 (4)	0.15207 (9)	0.0232 (3)	
H1B	0.2297	0.6219	0.1648	0.028*	
C2B	0.12167 (13)	0.58070 (4)	0.16165 (9)	0.0255 (3)	
C3B	0.10926 (13)	0.54597 (4)	0.14262 (9)	0.0253 (3)	
H3B	0.0402	0.5345	0.1490	0.030*	
C4B	0.19756 (13)	0.52809 (4)	0.11443 (9)	0.0249 (3)	
C5B	0.30432 (12)	0.54352 (4)	0.09843 (9)	0.0209 (3)	
C6B	0.30933 (12)	0.57958 (4)	0.12386 (9)	0.0214 (3)	
O1C	0.93330 (11)	0.49777 (4)	0.71717 (9)	0.0459 (3)	
O2C	0.95844 (11)	0.51289 (4)	0.59358 (10)	0.0504 (4)	
O3C	0.85836 (9)	0.57635 (3)	0.56438 (7)	0.0274 (2)	
O4C	0.70931 (11)	0.63065 (3)	0.55889 (8)	0.0392 (3)	
O5C	0.54488 (11)	0.60950 (4)	0.52653 (10)	0.0488 (4)	
O6C	0.42608 (9)	0.52706 (3)	0.72080 (8)	0.0357 (3)	
O7C	0.53457 (10)	0.48321 (3)	0.75141 (8)	0.0400 (3)	
N1C	0.90358 (11)	0.51276 (4)	0.65333 (9)	0.0307 (3)	
N2C	0.64000 (12)	0.60737 (4)	0.56060 (9)	0.0306 (3)	
N3C	0.51685 (11)	0.51198 (4)	0.72106 (8)	0.0287 (3)	
C1C	0.71259 (13)	0.51435 (4)	0.68752 (9)	0.0237 (3)	
H1C	0.7274	0.4937	0.7180	0.028*	
C2C	0.79609 (12)	0.53034 (4)	0.64799 (9)	0.0230 (3)	
C3C	0.78325 (12)	0.56224 (4)	0.60164 (9)	0.0217 (3)	
C4C	0.67031 (13)	0.57559 (4)	0.60354 (9)	0.0243 (3)	
C5C	0.58477 (13)	0.55929 (4)	0.63995 (9)	0.0255 (3)	
H5C	0.5116	0.5689	0.6361	0.031*	
C6C	0.60648 (12)	0.52896 (4)	0.68195 (9)	0.0241 (3)	
O1D	0.43842 (13)	0.41609 (4)	0.17945 (13)	0.0753 (6)	
O2D	0.45803 (12)	0.38759 (4)	0.06991 (9)	0.0483 (4)	
O3D	0.39104 (9)	0.32112 (3)	0.09394 (8)	0.0321 (3)	
O4D	0.27366 (16)	0.26401 (4)	0.11869 (13)	0.0756 (6)	
O5D	0.1088 (5)	0.2689 (2)	0.132 (2)	0.074 (6)	0.53 (5)
O5DA	0.1161 (9)	0.2685 (2)	0.1739 (14)	0.051 (3)	0.47 (5)
O6D	−0.04727 (10)	0.37181 (4)	0.23998 (8)	0.0417 (3)	
O7D	0.04676 (10)	0.41989 (3)	0.24429 (8)	0.0392 (3)	
N1D	0.41073 (12)	0.39325 (4)	0.13245 (10)	0.0335 (3)	
N2D	0.19780 (13)	0.28171 (4)	0.13810 (9)	0.0346 (3)	
N3D	0.03733 (11)	0.38876 (4)	0.22938 (8)	0.0310 (3)	
C1D	0.22787 (13)	0.38939 (4)	0.18260 (10)	0.0259 (3)	
H1D	0.2349	0.4132	0.1972	0.031*	
C2D	0.31297 (13)	0.37241 (4)	0.14767 (10)	0.0246 (3)	
C3D	0.31268 (12)	0.33593 (4)	0.12588 (9)	0.0237 (3)	
C4D	0.21039 (13)	0.31897 (4)	0.14756 (9)	0.0258 (3)	
C5D	0.12236 (13)	0.33613 (4)	0.17907 (10)	0.0271 (3)	
H5D	0.0559	0.3239	0.1889	0.033*	
C6D	0.13078 (13)	0.37097 (4)	0.19620 (10)	0.0266 (3)	
O1E	0.08024 (11)	0.32801 (3)	0.38651 (11)	0.0522 (4)	
O2E	0.00509 (10)	0.27786 (3)	0.36309 (9)	0.0387 (3)	
O3E	0.14077 (9)	0.23016 (3)	0.45016 (7)	0.0273 (2)	
O4E	0.28681 (12)	0.17609 (3)	0.42987 (9)	0.0453 (3)	
O5E	0.45292 (11)	0.19515 (4)	0.46414 (11)	0.0559 (4)	
O6E	0.56176 (10)	0.29016 (4)	0.29897 (8)	0.0429 (3)	
O7E	0.45539 (11)	0.33582 (4)	0.29169 (8)	0.0457 (3)	
N1E	0.08694 (11)	0.29654 (3)	0.37717 (8)	0.0274 (3)	
N2E	0.35637 (12)	0.19938 (4)	0.43450 (10)	0.0344 (3)	
N3E	0.47330 (12)	0.30510 (4)	0.30897 (8)	0.0337 (3)	
C1E	0.28020 (13)	0.30019 (4)	0.34811 (9)	0.0261 (3)	
H1E	0.2658	0.3233	0.3298	0.031*	
C2E	0.19720 (12)	0.28065 (4)	0.37890 (9)	0.0236 (3)	
C3E	0.21231 (12)	0.24610 (4)	0.41326 (9)	0.0221 (3)	
C4E	0.32526 (13)	0.23366 (4)	0.40462 (10)	0.0249 (3)	
C5E	0.40861 (13)	0.25255 (4)	0.37280 (9)	0.0265 (3)	
H5E	0.4814	0.2430	0.3705	0.032*	
C6E	0.38536 (13)	0.28565 (4)	0.34421 (9)	0.0270 (3)	
N4A	0.75790 (11)	0.09105 (3)	0.43652 (9)	0.0303 (3)	
N5A	0.69358 (11)	0.14260 (3)	0.39781 (8)	0.0244 (3)	
N6A	0.90342 (12)	0.23186 (3)	0.47233 (9)	0.0253 (3)	
H6AA	0.9724 (18)	0.2348 (5)	0.4655 (12)	0.032 (5)*	
H6AB	0.8939 (15)	0.2239 (5)	0.5237 (13)	0.030 (5)*	
H6AC	0.8659 (16)	0.2535 (5)	0.4655 (12)	0.034 (5)*	
C7A	0.66412 (14)	0.09844 (4)	0.47645 (10)	0.0295 (3)	
H7A	0.6323	0.0835	0.5146	0.035*	
C8A	0.77269 (13)	0.11818 (4)	0.38983 (11)	0.0276 (3)	
H8A	0.8319	0.1203	0.3547	0.033*	
C9A	0.62334 (13)	0.13017 (4)	0.45358 (10)	0.0279 (3)	
H9A	0.5595	0.1414	0.4723	0.033*	
C10A	0.68255 (14)	0.17569 (4)	0.35409 (10)	0.0284 (3)	
H10A	0.6025	0.1801	0.3383	0.034*	
H10B	0.7224	0.1742	0.3035	0.034*	
C11A	0.72981 (13)	0.20599 (4)	0.40554 (11)	0.0271 (3)	
H11A	0.7011	0.2281	0.3812	0.033*	
H11B	0.7038	0.2042	0.4612	0.033*	
C12A	0.85708 (13)	0.20646 (4)	0.41082 (11)	0.0277 (3)	
H12A	0.8856	0.1830	0.4255	0.033*	
H12B	0.8830	0.2125	0.3567	0.033*	
N4B	0.73741 (12)	0.39002 (3)	0.09045 (10)	0.0337 (3)	
N5B	0.80676 (11)	0.44049 (3)	0.13254 (8)	0.0250 (3)	
N6B	0.61280 (11)	0.53074 (3)	0.04321 (9)	0.0245 (3)	
H6BA	0.6219 (15)	0.5212 (5)	−0.0074 (12)	0.029 (5)*	
H6BB	0.5412 (18)	0.5353 (5)	0.0484 (12)	0.040 (6)*	
H6BC	0.6517 (15)	0.5514 (5)	0.0468 (11)	0.029 (5)*	
C7B	0.83655 (14)	0.39557 (4)	0.05633 (11)	0.0305 (4)	
H7B	0.8699	0.3800	0.0204	0.037*	
C8B	0.72217 (14)	0.41758 (4)	0.13569 (11)	0.0309 (4)	
H8B	0.6592	0.4209	0.1668	0.037*	
C9B	0.88023 (13)	0.42658 (4)	0.08124 (10)	0.0280 (3)	
H9B	0.9481	0.4367	0.0662	0.034*	
C10B	0.81931 (14)	0.47403 (4)	0.17389 (10)	0.0288 (3)	
H10C	0.8990	0.4776	0.1924	0.035*	
H10D	0.7757	0.4739	0.2228	0.035*	
C11B	0.77981 (13)	0.50414 (4)	0.11863 (10)	0.0265 (3)	
H11C	0.8114	0.5261	0.1416	0.032*	
H11D	0.8080	0.5008	0.0640	0.032*	
C12B	0.65295 (13)	0.50676 (4)	0.11003 (10)	0.0269 (3)	
H12C	0.6209	0.4834	0.0989	0.032*	
H12D	0.6260	0.5151	0.1621	0.032*	
N4C	0.77635 (12)	0.29279 (3)	0.44507 (10)	0.0333 (3)	
N5C	0.71008 (11)	0.34335 (3)	0.40054 (9)	0.0267 (3)	
N6C	0.91077 (12)	0.43386 (3)	0.47087 (9)	0.0238 (3)	
H6CA	0.9797 (17)	0.4372 (5)	0.4660 (11)	0.027 (5)*	
H6CB	0.9011 (15)	0.4268 (5)	0.5215 (12)	0.026 (5)*	
H6CC	0.8729 (16)	0.4547 (5)	0.4643 (12)	0.034 (5)*	
C7C	0.68308 (15)	0.30101 (4)	0.48517 (11)	0.0326 (4)	
H7C	0.6523	0.2870	0.5255	0.039*	
C8C	0.64165 (14)	0.33207 (4)	0.45851 (11)	0.0310 (4)	
H8C	0.5779	0.3437	0.4764	0.037*	
C9C	0.78943 (14)	0.31884 (4)	0.39487 (11)	0.0302 (4)	
H9C	0.8478	0.3202	0.3589	0.036*	
C10C	0.69767 (14)	0.37533 (4)	0.35294 (11)	0.0300 (4)	
H10E	0.6176	0.3788	0.3355	0.036*	
H10F	0.7390	0.3730	0.3032	0.036*	
C11C	0.74117 (13)	0.40700 (4)	0.40111 (11)	0.0278 (3)	
H11E	0.7123	0.4283	0.3733	0.033*	
H11F	0.7129	0.4064	0.4562	0.033*	
C12C	0.86828 (13)	0.40808 (4)	0.40902 (10)	0.0273 (3)	
H12E	0.8975	0.3848	0.4247	0.033*	
H12F	0.8961	0.4141	0.3555	0.033*	
N4D	0.22010 (12)	0.50702 (3)	0.56986 (9)	0.0317 (3)	
N5D	0.28930 (11)	0.46050 (3)	0.63391 (8)	0.0258 (3)	
N6D	0.11287 (12)	0.36876 (3)	0.55463 (9)	0.0233 (3)	
H6DA	0.0399 (17)	0.3640 (5)	0.5526 (11)	0.029 (5)*	
H6DB	0.1314 (16)	0.3766 (5)	0.5062 (13)	0.035 (5)*	
H6DC	0.1483 (15)	0.3488 (5)	0.5657 (11)	0.028 (5)*	
C7D	0.36432 (14)	0.47005 (4)	0.57872 (10)	0.0298 (3)	
H7D	0.4329	0.4589	0.5693	0.036*	
C8D	0.20416 (13)	0.48345 (4)	0.62573 (10)	0.0277 (3)	
H8D	0.1403	0.4827	0.6567	0.033*	
C9D	0.32091 (14)	0.49861 (4)	0.54037 (11)	0.0318 (4)	
H9D	0.3553	0.5111	0.4990	0.038*	
C10D	0.29807 (14)	0.43027 (4)	0.68810 (10)	0.0289 (3)	
H10G	0.2483	0.4336	0.7330	0.035*	
H10H	0.3758	0.4284	0.7126	0.035*	
C11D	0.26634 (13)	0.39670 (4)	0.64306 (10)	0.0271 (3)	
H11G	0.3086	0.3950	0.5937	0.033*	
H11H	0.2875	0.3767	0.6787	0.033*	
C12D	0.14198 (13)	0.39512 (4)	0.61840 (10)	0.0282 (3)	
H12G	0.1013	0.3898	0.6672	0.034*	
H12H	0.1167	0.4182	0.5979	0.034*	
N4E	0.23642 (12)	0.30890 (4)	0.59484 (10)	0.0341 (3)	
N5E	0.30112 (11)	0.26041 (3)	0.65175 (8)	0.0264 (3)	
N6E	0.11830 (12)	0.17068 (3)	0.55652 (9)	0.0244 (3)	
H6EA	0.0441 (19)	0.1656 (5)	0.5537 (13)	0.040 (6)*	
H6EB	0.1349 (16)	0.1796 (5)	0.5102 (13)	0.031 (5)*	
H6EC	0.1545 (17)	0.1495 (5)	0.5656 (12)	0.038 (5)*	
C7E	0.34032 (15)	0.30216 (4)	0.56911 (11)	0.0334 (4)	
H7E	0.3782	0.3163	0.5326	0.040*	
C8E	0.21567 (14)	0.28319 (4)	0.64442 (11)	0.0308 (4)	
H8E	0.1488	0.2810	0.6716	0.037*	
C9E	0.38128 (14)	0.27238 (4)	0.60320 (11)	0.0314 (4)	
H9E	0.4514	0.2619	0.5952	0.038*	
C10E	0.30688 (15)	0.22809 (4)	0.69875 (10)	0.0309 (4)	
H10I	0.2562	0.2298	0.7436	0.037*	
H10J	0.3840	0.2249	0.7235	0.037*	
C11E	0.27412 (14)	0.19658 (4)	0.64595 (11)	0.0289 (3)	
H11I	0.3144	0.1974	0.5955	0.035*	
H11J	0.2970	0.1751	0.6759	0.035*	
C12E	0.14910 (13)	0.19551 (4)	0.62346 (10)	0.0285 (3)	
H12I	0.1101	0.1889	0.6722	0.034*	
H12J	0.1233	0.2190	0.6066	0.034*	

### Atomic displacement parameters (Å^2^)

**Table:**

	*U*^11^	*U*^22^	*U*^33^	*U*^12^	*U*^13^	*U*^23^
O1A	0.0449 (8)	0.0491 (9)	0.0694 (10)	0.0187 (7)	0.0076 (7)	0.0317 (8)
O2A	0.0382 (7)	0.0381 (7)	0.0701 (10)	0.0121 (6)	0.0267 (7)	0.0157 (7)
O3A	0.0234 (6)	0.0277 (6)	0.0385 (7)	0.0032 (4)	0.0086 (5)	0.0102 (5)
O4A	0.0497 (8)	0.0259 (7)	0.0754 (10)	−0.0009 (6)	0.0289 (7)	0.0023 (6)
O5A	0.0416 (8)	0.0411 (8)	0.1072 (14)	0.0184 (7)	−0.0008 (8)	0.0171 (9)
O6A	0.0233 (6)	0.0594 (9)	0.0435 (7)	0.0038 (6)	0.0105 (5)	0.0068 (6)
O7A	0.0383 (7)	0.0441 (8)	0.0531 (8)	−0.0064 (6)	0.0156 (6)	0.0134 (6)
N1A	0.0243 (7)	0.0262 (7)	0.0490 (9)	0.0023 (6)	0.0077 (6)	0.0102 (6)
N2A	0.0376 (8)	0.0271 (7)	0.0323 (8)	0.0070 (6)	0.0076 (6)	0.0011 (6)
N3A	0.0255 (7)	0.0429 (9)	0.0275 (7)	−0.0041 (6)	0.0058 (6)	0.0023 (6)
C1A	0.0260 (8)	0.0261 (8)	0.0257 (8)	−0.0018 (6)	0.0018 (6)	0.0033 (6)
C2A	0.0202 (7)	0.0255 (8)	0.0278 (8)	0.0017 (6)	0.0018 (6)	0.0035 (6)
C3A	0.0203 (7)	0.0244 (8)	0.0221 (7)	−0.0002 (6)	−0.0003 (6)	0.0012 (6)
C4A	0.0261 (8)	0.0250 (8)	0.0226 (7)	0.0023 (6)	0.0011 (6)	0.0014 (6)
C5A	0.0216 (8)	0.0341 (9)	0.0227 (8)	0.0043 (6)	0.0026 (6)	−0.0016 (6)
C6A	0.0219 (8)	0.0336 (9)	0.0229 (8)	−0.0027 (6)	0.0045 (6)	−0.0003 (6)
O1B	0.0373 (7)	0.0221 (6)	0.0538 (8)	−0.0069 (5)	0.0042 (6)	0.0045 (5)
O2B	0.0200 (6)	0.0304 (6)	0.0647 (9)	−0.0008 (5)	0.0011 (5)	−0.0070 (6)
O3B	0.0235 (5)	0.0231 (5)	0.0322 (6)	−0.0030 (4)	0.0061 (4)	−0.0062 (4)
O4B	0.0530 (8)	0.0236 (6)	0.0551 (8)	−0.0036 (6)	0.0182 (7)	−0.0002 (6)
O5B	0.0431 (8)	0.0511 (9)	0.0791 (11)	−0.0284 (7)	0.0180 (8)	−0.0235 (8)
O6B	0.0230 (6)	0.0589 (8)	0.0369 (7)	0.0004 (6)	0.0098 (5)	−0.0014 (6)
O7B	0.0309 (6)	0.0438 (8)	0.0407 (7)	0.0103 (5)	−0.0015 (5)	−0.0134 (6)
N1B	0.0236 (7)	0.0428 (8)	0.0231 (7)	0.0060 (6)	0.0000 (5)	−0.0044 (6)
N2B	0.0387 (8)	0.0298 (8)	0.0325 (8)	−0.0143 (6)	0.0147 (6)	−0.0058 (6)
N3B	0.0232 (7)	0.0208 (6)	0.0295 (7)	−0.0022 (5)	0.0050 (5)	−0.0039 (5)
C1B	0.0250 (8)	0.0230 (7)	0.0212 (7)	0.0012 (6)	−0.0017 (6)	−0.0012 (6)
C2B	0.0207 (7)	0.0349 (9)	0.0208 (7)	0.0031 (6)	0.0014 (6)	−0.0009 (6)
C3B	0.0214 (7)	0.0341 (9)	0.0206 (7)	−0.0067 (6)	0.0031 (6)	0.0010 (6)
C4B	0.0278 (8)	0.0252 (8)	0.0220 (7)	−0.0067 (6)	0.0037 (6)	−0.0015 (6)
C5B	0.0199 (7)	0.0226 (7)	0.0201 (7)	−0.0010 (6)	0.0011 (5)	0.0007 (6)
C6B	0.0195 (7)	0.0222 (7)	0.0224 (7)	−0.0029 (6)	0.0013 (6)	0.0006 (6)
O1C	0.0330 (7)	0.0508 (8)	0.0523 (8)	0.0039 (6)	−0.0072 (6)	0.0256 (7)
O2C	0.0337 (7)	0.0504 (8)	0.0702 (10)	0.0179 (6)	0.0259 (7)	0.0294 (7)
O3C	0.0223 (5)	0.0256 (6)	0.0351 (6)	0.0003 (4)	0.0075 (5)	0.0091 (5)
O4C	0.0440 (7)	0.0218 (6)	0.0542 (8)	0.0026 (5)	0.0191 (6)	0.0055 (5)
O5C	0.0311 (7)	0.0484 (8)	0.0668 (10)	0.0159 (6)	0.0038 (6)	0.0232 (7)
O6C	0.0219 (6)	0.0472 (7)	0.0388 (7)	−0.0020 (5)	0.0091 (5)	0.0035 (6)
O7C	0.0344 (7)	0.0417 (7)	0.0449 (7)	−0.0061 (5)	0.0092 (6)	0.0163 (6)
N1C	0.0206 (7)	0.0277 (7)	0.0439 (8)	−0.0014 (5)	0.0019 (6)	0.0150 (6)
N2C	0.0305 (8)	0.0259 (7)	0.0371 (8)	0.0084 (6)	0.0140 (6)	0.0057 (6)
N3C	0.0249 (7)	0.0363 (8)	0.0253 (7)	−0.0066 (6)	0.0051 (5)	0.0024 (6)
C1C	0.0250 (8)	0.0239 (7)	0.0219 (7)	−0.0031 (6)	0.0010 (6)	0.0031 (6)
C2C	0.0202 (7)	0.0249 (8)	0.0236 (7)	−0.0009 (6)	0.0004 (6)	0.0019 (6)
C3C	0.0213 (7)	0.0227 (7)	0.0213 (7)	−0.0017 (6)	0.0022 (6)	0.0017 (6)
C4C	0.0267 (8)	0.0215 (7)	0.0249 (8)	0.0021 (6)	0.0038 (6)	0.0015 (6)
C5C	0.0215 (7)	0.0290 (8)	0.0267 (8)	0.0024 (6)	0.0057 (6)	−0.0011 (6)
C6C	0.0222 (8)	0.0288 (8)	0.0218 (7)	−0.0051 (6)	0.0054 (6)	0.0000 (6)
O1D	0.0514 (9)	0.0660 (11)	0.1123 (15)	−0.0325 (8)	0.0321 (10)	−0.0561 (11)
O2D	0.0426 (8)	0.0415 (8)	0.0634 (10)	−0.0059 (6)	0.0214 (7)	−0.0024 (7)
O3D	0.0250 (6)	0.0276 (6)	0.0445 (7)	−0.0016 (5)	0.0075 (5)	−0.0118 (5)
O4D	0.0878 (13)	0.0257 (7)	0.1225 (16)	−0.0057 (8)	0.0709 (12)	−0.0080 (8)
O5D	0.038 (2)	0.045 (2)	0.141 (16)	−0.0225 (16)	0.015 (4)	−0.031 (5)
O5DA	0.045 (2)	0.035 (2)	0.075 (7)	−0.0157 (16)	0.025 (3)	−0.008 (3)
O6D	0.0262 (6)	0.0556 (8)	0.0445 (8)	−0.0018 (6)	0.0111 (5)	−0.0065 (6)
O7D	0.0333 (7)	0.0399 (7)	0.0446 (7)	0.0085 (5)	0.0031 (5)	−0.0130 (6)
N1D	0.0243 (7)	0.0248 (7)	0.0523 (9)	−0.0015 (5)	0.0087 (6)	−0.0080 (6)
N2D	0.0384 (9)	0.0290 (8)	0.0369 (8)	−0.0069 (6)	0.0062 (7)	−0.0043 (6)
N3D	0.0245 (7)	0.0423 (9)	0.0260 (7)	0.0038 (6)	0.0007 (5)	−0.0049 (6)
C1D	0.0253 (8)	0.0242 (8)	0.0280 (8)	0.0015 (6)	−0.0001 (6)	−0.0052 (6)
C2D	0.0211 (7)	0.0244 (8)	0.0283 (8)	−0.0016 (6)	0.0015 (6)	−0.0022 (6)
C3D	0.0211 (7)	0.0251 (8)	0.0246 (8)	0.0003 (6)	−0.0004 (6)	−0.0040 (6)
C4D	0.0271 (8)	0.0264 (8)	0.0238 (8)	−0.0030 (6)	0.0000 (6)	−0.0025 (6)
C5D	0.0220 (8)	0.0343 (9)	0.0252 (8)	−0.0041 (6)	0.0025 (6)	−0.0005 (6)
C6D	0.0227 (8)	0.0334 (9)	0.0237 (8)	0.0028 (6)	0.0020 (6)	−0.0027 (6)
O1E	0.0381 (8)	0.0269 (7)	0.0914 (12)	0.0065 (5)	0.0036 (7)	0.0034 (7)
O2E	0.0224 (6)	0.0378 (7)	0.0552 (8)	−0.0012 (5)	−0.0018 (5)	0.0052 (6)
O3E	0.0225 (6)	0.0262 (6)	0.0341 (6)	−0.0002 (4)	0.0068 (5)	0.0058 (5)
O4E	0.0464 (8)	0.0228 (6)	0.0692 (10)	−0.0008 (6)	0.0221 (7)	−0.0001 (6)
O5E	0.0317 (7)	0.0446 (8)	0.0922 (12)	0.0163 (6)	0.0089 (7)	0.0199 (8)
O6E	0.0254 (7)	0.0687 (9)	0.0358 (7)	−0.0100 (6)	0.0100 (5)	0.0017 (6)
O7E	0.0402 (7)	0.0501 (8)	0.0457 (8)	−0.0185 (6)	−0.0040 (6)	0.0210 (7)
N1E	0.0236 (7)	0.0273 (7)	0.0315 (7)	−0.0001 (5)	0.0020 (5)	0.0073 (6)
N2E	0.0322 (8)	0.0280 (7)	0.0449 (9)	0.0089 (6)	0.0164 (7)	0.0009 (6)
N3E	0.0282 (8)	0.0489 (9)	0.0234 (7)	−0.0141 (6)	−0.0014 (6)	0.0073 (6)
C1E	0.0277 (8)	0.0277 (8)	0.0222 (8)	−0.0055 (6)	−0.0019 (6)	0.0045 (6)
C2E	0.0203 (7)	0.0263 (8)	0.0240 (7)	−0.0010 (6)	0.0003 (6)	0.0008 (6)
C3E	0.0219 (7)	0.0227 (7)	0.0219 (7)	−0.0018 (6)	0.0022 (6)	−0.0019 (6)
C4E	0.0247 (8)	0.0240 (8)	0.0266 (8)	0.0001 (6)	0.0060 (6)	−0.0009 (6)
C5E	0.0208 (7)	0.0341 (9)	0.0251 (8)	−0.0004 (6)	0.0051 (6)	−0.0037 (6)
C6E	0.0239 (8)	0.0365 (9)	0.0206 (7)	−0.0096 (6)	0.0017 (6)	0.0015 (6)
N4A	0.0278 (7)	0.0204 (7)	0.0421 (8)	0.0044 (5)	−0.0006 (6)	0.0008 (6)
N5A	0.0233 (6)	0.0186 (6)	0.0311 (7)	0.0019 (5)	0.0003 (5)	0.0001 (5)
N6A	0.0192 (7)	0.0209 (7)	0.0364 (8)	0.0005 (5)	0.0060 (6)	0.0041 (6)
C7A	0.0299 (9)	0.0250 (8)	0.0333 (9)	0.0003 (6)	0.0009 (7)	0.0036 (7)
C8A	0.0236 (8)	0.0211 (8)	0.0384 (9)	0.0033 (6)	0.0039 (7)	−0.0018 (6)
C9A	0.0251 (8)	0.0263 (8)	0.0325 (9)	0.0042 (6)	0.0041 (6)	−0.0012 (7)
C10A	0.0293 (8)	0.0214 (8)	0.0337 (9)	0.0026 (6)	−0.0021 (7)	0.0042 (6)
C11A	0.0240 (8)	0.0193 (7)	0.0382 (9)	0.0031 (6)	0.0035 (7)	0.0011 (6)
C12A	0.0243 (8)	0.0225 (8)	0.0372 (9)	0.0026 (6)	0.0080 (7)	−0.0010 (7)
N4B	0.0290 (7)	0.0199 (7)	0.0516 (9)	−0.0043 (5)	−0.0009 (6)	0.0004 (6)
N5B	0.0234 (7)	0.0219 (6)	0.0294 (7)	−0.0031 (5)	−0.0003 (5)	0.0008 (5)
N6B	0.0204 (7)	0.0190 (6)	0.0347 (8)	−0.0027 (5)	0.0064 (6)	−0.0037 (5)
C7B	0.0298 (9)	0.0260 (8)	0.0352 (9)	0.0004 (6)	−0.0003 (7)	−0.0016 (7)
C8B	0.0253 (8)	0.0244 (8)	0.0435 (10)	−0.0037 (6)	0.0058 (7)	0.0037 (7)
C9B	0.0238 (8)	0.0267 (8)	0.0338 (9)	−0.0033 (6)	0.0027 (6)	−0.0003 (7)
C10B	0.0299 (8)	0.0268 (8)	0.0294 (8)	−0.0037 (6)	−0.0009 (6)	−0.0042 (7)
C11B	0.0252 (8)	0.0204 (7)	0.0343 (9)	−0.0044 (6)	0.0048 (6)	−0.0029 (6)
C12B	0.0248 (8)	0.0238 (8)	0.0329 (8)	−0.0039 (6)	0.0078 (6)	0.0002 (6)
N4C	0.0319 (8)	0.0211 (7)	0.0462 (9)	0.0051 (6)	−0.0016 (6)	−0.0013 (6)
N5C	0.0248 (7)	0.0193 (6)	0.0353 (7)	0.0032 (5)	−0.0013 (5)	−0.0022 (5)
N6C	0.0186 (7)	0.0226 (7)	0.0304 (7)	0.0003 (5)	0.0034 (5)	0.0042 (5)
C7C	0.0349 (9)	0.0269 (8)	0.0358 (9)	0.0014 (7)	0.0013 (7)	0.0009 (7)
C8C	0.0284 (8)	0.0283 (8)	0.0363 (9)	0.0061 (7)	0.0026 (7)	−0.0032 (7)
C9C	0.0264 (8)	0.0210 (8)	0.0433 (10)	0.0021 (6)	0.0020 (7)	−0.0036 (7)
C10C	0.0299 (8)	0.0232 (8)	0.0358 (9)	0.0019 (6)	−0.0047 (7)	0.0017 (7)
C11C	0.0226 (8)	0.0206 (8)	0.0399 (9)	0.0035 (6)	0.0002 (7)	−0.0001 (7)
C12C	0.0248 (8)	0.0250 (8)	0.0325 (8)	0.0034 (6)	0.0047 (6)	−0.0012 (6)
N4D	0.0304 (7)	0.0217 (7)	0.0421 (8)	0.0027 (5)	−0.0029 (6)	−0.0012 (6)
N5D	0.0236 (7)	0.0222 (7)	0.0309 (7)	0.0034 (5)	−0.0024 (5)	−0.0023 (5)
N6D	0.0218 (7)	0.0198 (7)	0.0290 (7)	0.0016 (5)	0.0049 (5)	0.0026 (5)
C7D	0.0245 (8)	0.0292 (8)	0.0355 (9)	0.0028 (6)	0.0018 (7)	−0.0033 (7)
C8D	0.0231 (8)	0.0232 (8)	0.0369 (9)	0.0029 (6)	0.0020 (6)	−0.0048 (7)
C9D	0.0315 (9)	0.0294 (9)	0.0342 (9)	−0.0021 (7)	0.0017 (7)	−0.0004 (7)
C10D	0.0286 (8)	0.0279 (8)	0.0294 (8)	0.0044 (6)	−0.0036 (6)	0.0012 (7)
C11D	0.0258 (8)	0.0236 (8)	0.0316 (8)	0.0056 (6)	−0.0009 (6)	0.0014 (6)
C12D	0.0258 (8)	0.0275 (8)	0.0317 (8)	0.0036 (6)	0.0051 (6)	−0.0048 (7)
N4E	0.0329 (8)	0.0224 (7)	0.0462 (9)	0.0037 (6)	−0.0022 (6)	0.0007 (6)
N5E	0.0263 (7)	0.0217 (6)	0.0307 (7)	0.0037 (5)	−0.0018 (5)	−0.0007 (5)
N6E	0.0218 (7)	0.0200 (7)	0.0319 (8)	0.0015 (5)	0.0056 (6)	0.0022 (5)
C7E	0.0328 (9)	0.0277 (8)	0.0395 (10)	0.0000 (7)	0.0014 (7)	0.0027 (7)
C8E	0.0286 (9)	0.0238 (8)	0.0400 (10)	0.0042 (6)	0.0027 (7)	−0.0040 (7)
C9E	0.0251 (8)	0.0305 (9)	0.0386 (9)	0.0033 (7)	0.0025 (7)	−0.0006 (7)
C10E	0.0345 (9)	0.0277 (8)	0.0295 (9)	0.0030 (7)	−0.0049 (7)	0.0038 (7)
C11E	0.0286 (9)	0.0232 (8)	0.0346 (9)	0.0043 (6)	−0.0008 (7)	0.0027 (7)
C12E	0.0280 (8)	0.0273 (8)	0.0307 (9)	0.0032 (6)	0.0056 (7)	−0.0030 (7)

### Geometric parameters (Å, º)

**Table:**

O1A—N1A	1.227 (2)	N6A—H6AC	0.95 (2)
O2A—N1A	1.224 (2)	N6A—C12A	1.485 (2)
O3A—C3A	1.2448 (18)	C7A—H7A	0.9500
O4A—N2A	1.2268 (19)	C7A—C9A	1.359 (2)
O5A—N2A	1.2139 (19)	C8A—H8A	0.9500
O6A—N3A	1.2322 (19)	C9A—H9A	0.9500
O7A—N3A	1.223 (2)	C10A—H10A	0.9900
N1A—C2A	1.459 (2)	C10A—H10B	0.9900
N2A—C4A	1.449 (2)	C10A—C11A	1.525 (2)
N3A—C6A	1.449 (2)	C11A—H11A	0.9900
C1A—H1A	0.9500	C11A—H11B	0.9900
C1A—C2A	1.373 (2)	C11A—C12A	1.521 (2)
C1A—C6A	1.396 (2)	C12A—H12A	0.9900
C2A—C3A	1.452 (2)	C12A—H12B	0.9900
C3A—C4A	1.453 (2)	N4B—C7B	1.367 (2)
C4A—C5A	1.376 (2)	N4B—C8B	1.316 (2)
C5A—H5A	0.9500	N5B—C8B	1.348 (2)
C5A—C6A	1.376 (2)	N5B—C9B	1.371 (2)
O1B—N3B	1.2231 (17)	N5B—C10B	1.462 (2)
O2B—N3B	1.2270 (17)	N6B—H6BA	0.92 (2)
O3B—C5B	1.2451 (18)	N6B—H6BB	0.89 (2)
O4B—N2B	1.225 (2)	N6B—H6BC	0.92 (2)
O5B—N2B	1.2291 (19)	N6B—C12B	1.487 (2)
O6B—N1B	1.2295 (19)	C7B—H7B	0.9500
O7B—N1B	1.2298 (19)	C7B—C9B	1.355 (2)
N1B—C2B	1.447 (2)	C8B—H8B	0.9500
N2B—C4B	1.453 (2)	C9B—H9B	0.9500
N3B—C6B	1.4567 (18)	C10B—H10C	0.9900
C1B—H1B	0.9500	C10B—H10D	0.9900
C1B—C2B	1.391 (2)	C10B—C11B	1.526 (2)
C1B—C6B	1.371 (2)	C11B—H11C	0.9900
C2B—C3B	1.380 (2)	C11B—H11D	0.9900
C3B—H3B	0.9500	C11B—C12B	1.520 (2)
C3B—C4B	1.371 (2)	C12B—H12C	0.9900
C4B—C5B	1.452 (2)	C12B—H12D	0.9900
C5B—C6B	1.451 (2)	N4C—C7C	1.375 (2)
O1C—N1C	1.2267 (18)	N4C—C9C	1.315 (2)
O2C—N1C	1.2206 (19)	N5C—C8C	1.372 (2)
O3C—C3C	1.2492 (18)	N5C—C9C	1.348 (2)
O4C—N2C	1.2238 (18)	N5C—C10C	1.461 (2)
O5C—N2C	1.235 (2)	N6C—H6CA	0.85 (2)
O6C—N3C	1.2329 (18)	N6C—H6CB	0.89 (2)
O7C—N3C	1.2273 (19)	N6C—H6CC	0.92 (2)
N1C—C2C	1.4523 (19)	N6C—C12C	1.484 (2)
N2C—C4C	1.446 (2)	C7C—H7C	0.9500
N3C—C6C	1.4474 (19)	C7C—C8C	1.356 (2)
C1C—H1C	0.9500	C8C—H8C	0.9500
C1C—C2C	1.377 (2)	C9C—H9C	0.9500
C1C—C6C	1.387 (2)	C10C—H10E	0.9900
C2C—C3C	1.448 (2)	C10C—H10F	0.9900
C3C—C4C	1.450 (2)	C10C—C11C	1.525 (2)
C4C—C5C	1.375 (2)	C11C—H11E	0.9900
C5C—H5C	0.9500	C11C—H11F	0.9900
C5C—C6C	1.372 (2)	C11C—C12C	1.520 (2)
O1D—N1D	1.201 (2)	C12C—H12E	0.9900
O2D—N1D	1.229 (2)	C12C—H12F	0.9900
O3D—C3D	1.2469 (18)	N4D—C8D	1.315 (2)
O4D—N2D	1.198 (2)	N4D—C9D	1.373 (2)
O5D—N2D	1.172 (6)	N5D—C7D	1.373 (2)
O5DA—N2D	1.284 (6)	N5D—C8D	1.3493 (19)
O6D—N3D	1.2288 (19)	N5D—C10D	1.465 (2)
O7D—N3D	1.2276 (19)	N6D—H6DA	0.89 (2)
N1D—C2D	1.457 (2)	N6D—H6DB	0.89 (2)
N2D—C4D	1.451 (2)	N6D—H6DC	0.89 (2)
N3D—C6D	1.452 (2)	N6D—C12D	1.482 (2)
C1D—H1D	0.9500	C7D—H7D	0.9500
C1D—C2D	1.372 (2)	C7D—C9D	1.353 (2)
C1D—C6D	1.395 (2)	C8D—H8D	0.9500
C2D—C3D	1.450 (2)	C9D—H9D	0.9500
C3D—C4D	1.456 (2)	C10D—H10G	0.9900
C4D—C5D	1.378 (2)	C10D—H10H	0.9900
C5D—H5D	0.9500	C10D—C11D	1.524 (2)
C5D—C6D	1.374 (2)	C11D—H11G	0.9900
O1E—N1E	1.2253 (18)	C11D—H11H	0.9900
O2E—N1E	1.2249 (18)	C11D—C12D	1.517 (2)
O3E—C3E	1.2472 (18)	C12D—H12G	0.9900
O4E—N2E	1.2234 (19)	C12D—H12H	0.9900
O5E—N2E	1.233 (2)	N4E—C7E	1.369 (2)
O6E—N3E	1.228 (2)	N4E—C8E	1.317 (2)
O7E—N3E	1.232 (2)	N5E—C8E	1.347 (2)
N1E—C2E	1.4546 (19)	N5E—C9E	1.372 (2)
N2E—C4E	1.448 (2)	N5E—C10E	1.464 (2)
N3E—C6E	1.448 (2)	N6E—H6EA	0.91 (2)
C1E—H1E	0.9500	N6E—H6EB	0.87 (2)
C1E—C2E	1.373 (2)	N6E—H6EC	0.93 (2)
C1E—C6E	1.385 (2)	N6E—C12E	1.483 (2)
C2E—C3E	1.452 (2)	C7E—H7E	0.9500
C3E—C4E	1.453 (2)	C7E—C9E	1.353 (2)
C4E—C5E	1.370 (2)	C8E—H8E	0.9500
C5E—H5E	0.9500	C9E—H9E	0.9500
C5E—C6E	1.380 (2)	C10E—H10I	0.9900
N4A—C7A	1.372 (2)	C10E—H10J	0.9900
N4A—C8A	1.316 (2)	C10E—C11E	1.526 (2)
N5A—C8A	1.3485 (19)	C11E—H11I	0.9900
N5A—C9A	1.374 (2)	C11E—H11J	0.9900
N5A—C10A	1.4645 (19)	C11E—C12E	1.517 (2)
N6A—H6AA	0.85 (2)	C12E—H12I	0.9900
N6A—H6AB	0.91 (2)	C12E—H12J	0.9900
			
O1A—N1A—C2A	117.71 (14)	C11A—C10A—H10B	109.2
O2A—N1A—O1A	123.60 (15)	C10A—C11A—H11A	109.3
O2A—N1A—C2A	118.68 (14)	C10A—C11A—H11B	109.3
O4A—N2A—C4A	119.55 (14)	H11A—C11A—H11B	107.9
O5A—N2A—O4A	121.60 (15)	C12A—C11A—C10A	111.75 (13)
O5A—N2A—C4A	118.85 (15)	C12A—C11A—H11A	109.3
O6A—N3A—C6A	117.84 (15)	C12A—C11A—H11B	109.3
O7A—N3A—O6A	123.80 (14)	N6A—C12A—C11A	111.71 (13)
O7A—N3A—C6A	118.35 (14)	N6A—C12A—H12A	109.3
C2A—C1A—H1A	120.6	N6A—C12A—H12B	109.3
C2A—C1A—C6A	118.77 (15)	C11A—C12A—H12A	109.3
C6A—C1A—H1A	120.6	C11A—C12A—H12B	109.3
C1A—C2A—N1A	116.22 (14)	H12A—C12A—H12B	107.9
C1A—C2A—C3A	124.90 (14)	C8B—N4B—C7B	105.42 (14)
C3A—C2A—N1A	118.87 (13)	C8B—N5B—C9B	106.63 (13)
O3A—C3A—C2A	124.92 (14)	C8B—N5B—C10B	127.79 (14)
O3A—C3A—C4A	124.07 (14)	C9B—N5B—C10B	125.57 (13)
C2A—C3A—C4A	111.01 (13)	H6BA—N6B—H6BB	110.2 (17)
N2A—C4A—C3A	119.08 (14)	H6BA—N6B—H6BC	108.0 (16)
C5A—C4A—N2A	116.44 (14)	H6BB—N6B—H6BC	108.1 (17)
C5A—C4A—C3A	124.48 (14)	C12B—N6B—H6BA	111.4 (11)
C4A—C5A—H5A	120.3	C12B—N6B—H6BB	108.3 (13)
C6A—C5A—C4A	119.37 (14)	C12B—N6B—H6BC	110.8 (12)
C6A—C5A—H5A	120.3	N4B—C7B—H7B	125.0
C1A—C6A—N3A	118.97 (15)	C9B—C7B—N4B	110.00 (15)
C5A—C6A—N3A	120.01 (14)	C9B—C7B—H7B	125.0
C5A—C6A—C1A	121.00 (14)	N4B—C8B—N5B	111.75 (15)
O6B—N1B—O7B	123.70 (14)	N4B—C8B—H8B	124.1
O6B—N1B—C2B	118.19 (14)	N5B—C8B—H8B	124.1
O7B—N1B—C2B	118.11 (14)	N5B—C9B—H9B	126.9
O4B—N2B—O5B	123.03 (15)	C7B—C9B—N5B	106.18 (14)
O4B—N2B—C4B	119.13 (14)	C7B—C9B—H9B	126.9
O5B—N2B—C4B	117.83 (15)	N5B—C10B—H10C	109.2
O1B—N3B—O2B	123.41 (13)	N5B—C10B—H10D	109.2
O1B—N3B—C6B	118.79 (13)	N5B—C10B—C11B	112.23 (13)
O2B—N3B—C6B	117.72 (13)	H10C—C10B—H10D	107.9
C2B—C1B—H1B	120.6	C11B—C10B—H10C	109.2
C6B—C1B—H1B	120.6	C11B—C10B—H10D	109.2
C6B—C1B—C2B	118.72 (14)	C10B—C11B—H11C	109.3
C1B—C2B—N1B	120.21 (15)	C10B—C11B—H11D	109.3
C3B—C2B—N1B	118.68 (14)	H11C—C11B—H11D	107.9
C3B—C2B—C1B	121.07 (14)	C12B—C11B—C10B	111.74 (13)
C2B—C3B—H3B	120.3	C12B—C11B—H11C	109.3
C4B—C3B—C2B	119.38 (14)	C12B—C11B—H11D	109.3
C4B—C3B—H3B	120.3	N6B—C12B—C11B	112.16 (13)
C3B—C4B—N2B	115.98 (14)	N6B—C12B—H12C	109.2
C3B—C4B—C5B	124.57 (14)	N6B—C12B—H12D	109.2
C5B—C4B—N2B	119.45 (14)	C11B—C12B—H12C	109.2
O3B—C5B—C4B	124.26 (14)	C11B—C12B—H12D	109.2
O3B—C5B—C6B	124.58 (13)	H12C—C12B—H12D	107.9
C6B—C5B—C4B	110.99 (13)	C9C—N4C—C7C	105.23 (14)
C1B—C6B—N3B	116.43 (13)	C8C—N5C—C10C	126.59 (14)
C1B—C6B—C5B	125.00 (14)	C9C—N5C—C8C	106.65 (14)
C5B—C6B—N3B	118.57 (13)	C9C—N5C—C10C	126.75 (15)
O1C—N1C—C2C	118.16 (14)	H6CA—N6C—H6CB	109.2 (17)
O2C—N1C—O1C	123.32 (14)	H6CA—N6C—H6CC	109.4 (17)
O2C—N1C—C2C	118.50 (14)	H6CB—N6C—H6CC	106.2 (16)
O4C—N2C—O5C	123.11 (14)	C12C—N6C—H6CA	109.0 (12)
O4C—N2C—C4C	119.00 (14)	C12C—N6C—H6CB	111.9 (12)
O5C—N2C—C4C	117.88 (14)	C12C—N6C—H6CC	111.1 (12)
O6C—N3C—C6C	117.94 (14)	N4C—C7C—H7C	125.1
O7C—N3C—O6C	123.59 (13)	C8C—C7C—N4C	109.87 (16)
O7C—N3C—C6C	118.46 (14)	C8C—C7C—H7C	125.1
C2C—C1C—H1C	120.6	N5C—C8C—H8C	126.9
C2C—C1C—C6C	118.77 (14)	C7C—C8C—N5C	106.29 (15)
C6C—C1C—H1C	120.6	C7C—C8C—H8C	126.9
C1C—C2C—N1C	115.76 (13)	N4C—C9C—N5C	111.97 (15)
C1C—C2C—C3C	125.07 (14)	N4C—C9C—H9C	124.0
C3C—C2C—N1C	119.16 (13)	N5C—C9C—H9C	124.0
O3C—C3C—C2C	125.04 (14)	N5C—C10C—H10E	109.1
O3C—C3C—C4C	124.28 (14)	N5C—C10C—H10F	109.1
C2C—C3C—C4C	110.67 (13)	N5C—C10C—C11C	112.30 (14)
N2C—C4C—C3C	119.42 (13)	H10E—C10C—H10F	107.9
C5C—C4C—N2C	115.46 (14)	C11C—C10C—H10E	109.1
C5C—C4C—C3C	125.02 (14)	C11C—C10C—H10F	109.1
C4C—C5C—H5C	120.4	C10C—C11C—H11E	109.3
C6C—C5C—C4C	119.16 (14)	C10C—C11C—H11F	109.3
C6C—C5C—H5C	120.4	H11E—C11C—H11F	108.0
C1C—C6C—N3C	119.58 (14)	C12C—C11C—C10C	111.63 (13)
C5C—C6C—N3C	119.24 (14)	C12C—C11C—H11E	109.3
C5C—C6C—C1C	121.18 (14)	C12C—C11C—H11F	109.3
O1D—N1D—O2D	123.01 (16)	N6C—C12C—C11C	111.62 (13)
O1D—N1D—C2D	118.78 (15)	N6C—C12C—H12E	109.3
O2D—N1D—C2D	118.13 (14)	N6C—C12C—H12F	109.3
O4D—N2D—O5DA	121.1 (4)	C11C—C12C—H12E	109.3
O4D—N2D—C4D	121.11 (15)	C11C—C12C—H12F	109.3
O5D—N2D—O4D	116.2 (6)	H12E—C12C—H12F	108.0
O5D—N2D—C4D	120.7 (4)	C8D—N4D—C9D	105.07 (14)
O5DA—N2D—C4D	114.8 (6)	C7D—N5D—C10D	126.36 (13)
O6D—N3D—C6D	117.99 (15)	C8D—N5D—C7D	106.60 (14)
O7D—N3D—O6D	123.75 (14)	C8D—N5D—C10D	126.98 (14)
O7D—N3D—C6D	118.26 (14)	H6DA—N6D—H6DB	110.1 (17)
C2D—C1D—H1D	120.7	H6DA—N6D—H6DC	106.3 (16)
C2D—C1D—C6D	118.59 (15)	H6DB—N6D—H6DC	109.1 (17)
C6D—C1D—H1D	120.7	C12D—N6D—H6DA	110.3 (12)
C1D—C2D—N1D	116.12 (14)	C12D—N6D—H6DB	109.7 (13)
C1D—C2D—C3D	125.24 (14)	C12D—N6D—H6DC	111.3 (12)
C3D—C2D—N1D	118.64 (13)	N5D—C7D—H7D	127.0
O3D—C3D—C2D	123.99 (14)	C9D—C7D—N5D	106.10 (14)
O3D—C3D—C4D	124.81 (14)	C9D—C7D—H7D	127.0
C2D—C3D—C4D	111.19 (13)	N4D—C8D—N5D	111.99 (15)
N2D—C4D—C3D	120.05 (14)	N4D—C8D—H8D	124.0
C5D—C4D—N2D	116.02 (14)	N5D—C8D—H8D	124.0
C5D—C4D—C3D	123.92 (15)	N4D—C9D—H9D	124.9
C4D—C5D—H5D	120.0	C7D—C9D—N4D	110.24 (15)
C6D—C5D—C4D	119.94 (15)	C7D—C9D—H9D	124.9
C6D—C5D—H5D	120.0	N5D—C10D—H10G	109.2
C1D—C6D—N3D	119.68 (15)	N5D—C10D—H10H	109.2
C5D—C6D—N3D	119.38 (14)	N5D—C10D—C11D	112.06 (13)
C5D—C6D—C1D	120.93 (15)	H10G—C10D—H10H	107.9
O1E—N1E—C2E	118.81 (13)	C11D—C10D—H10G	109.2
O2E—N1E—O1E	123.10 (14)	C11D—C10D—H10H	109.2
O2E—N1E—C2E	118.02 (13)	C10D—C11D—H11G	109.3
O4E—N2E—O5E	122.89 (15)	C10D—C11D—H11H	109.3
O4E—N2E—C4E	119.37 (15)	H11G—C11D—H11H	108.0
O5E—N2E—C4E	117.74 (15)	C12D—C11D—C10D	111.64 (13)
O6E—N3E—O7E	123.84 (15)	C12D—C11D—H11G	109.3
O6E—N3E—C6E	118.14 (15)	C12D—C11D—H11H	109.3
O7E—N3E—C6E	118.01 (15)	N6D—C12D—C11D	113.07 (13)
C2E—C1E—H1E	120.6	N6D—C12D—H12G	109.0
C2E—C1E—C6E	118.78 (15)	N6D—C12D—H12H	109.0
C6E—C1E—H1E	120.6	C11D—C12D—H12G	109.0
C1E—C2E—N1E	116.32 (14)	C11D—C12D—H12H	109.0
C1E—C2E—C3E	124.94 (14)	H12G—C12D—H12H	107.8
C3E—C2E—N1E	118.74 (13)	C8E—N4E—C7E	105.39 (14)
O3E—C3E—C2E	124.73 (14)	C8E—N5E—C9E	106.80 (14)
O3E—C3E—C4E	124.29 (14)	C8E—N5E—C10E	127.40 (15)
C2E—C3E—C4E	110.81 (13)	C9E—N5E—C10E	125.74 (14)
N2E—C4E—C3E	119.12 (13)	H6EA—N6E—H6EB	109.0 (18)
C5E—C4E—N2E	115.91 (14)	H6EA—N6E—H6EC	105.3 (17)
C5E—C4E—C3E	124.92 (15)	H6EB—N6E—H6EC	110.5 (17)
C4E—C5E—H5E	120.5	C12E—N6E—H6EA	111.4 (13)
C4E—C5E—C6E	119.08 (15)	C12E—N6E—H6EB	109.5 (13)
C6E—C5E—H5E	120.5	C12E—N6E—H6EC	111.0 (13)
C1E—C6E—N3E	120.04 (15)	N4E—C7E—H7E	124.9
C5E—C6E—N3E	118.65 (15)	C9E—C7E—N4E	110.10 (16)
C5E—C6E—C1E	121.31 (14)	C9E—C7E—H7E	124.9
C8A—N4A—C7A	105.28 (13)	N4E—C8E—N5E	111.62 (15)
C8A—N5A—C9A	106.91 (13)	N4E—C8E—H8E	124.2
C8A—N5A—C10A	126.75 (14)	N5E—C8E—H8E	124.2
C9A—N5A—C10A	126.32 (13)	N5E—C9E—H9E	127.0
H6AA—N6A—H6AB	110.8 (17)	C7E—C9E—N5E	106.09 (15)
H6AA—N6A—H6AC	109.0 (17)	C7E—C9E—H9E	127.0
H6AB—N6A—H6AC	108.3 (16)	N5E—C10E—H10I	109.2
C12A—N6A—H6AA	108.4 (13)	N5E—C10E—H10J	109.2
C12A—N6A—H6AB	110.0 (12)	N5E—C10E—C11E	112.11 (13)
C12A—N6A—H6AC	110.3 (12)	H10I—C10E—H10J	107.9
N4A—C7A—H7A	124.9	C11E—C10E—H10I	109.2
C9A—C7A—N4A	110.19 (15)	C11E—C10E—H10J	109.2
C9A—C7A—H7A	124.9	C10E—C11E—H11I	109.3
N4A—C8A—N5A	111.82 (14)	C10E—C11E—H11J	109.3
N4A—C8A—H8A	124.1	H11I—C11E—H11J	108.0
N5A—C8A—H8A	124.1	C12E—C11E—C10E	111.61 (13)
N5A—C9A—H9A	127.1	C12E—C11E—H11I	109.3
C7A—C9A—N5A	105.80 (14)	C12E—C11E—H11J	109.3
C7A—C9A—H9A	127.1	N6E—C12E—C11E	112.69 (13)
N5A—C10A—H10A	109.2	N6E—C12E—H12I	109.1
N5A—C10A—H10B	109.2	N6E—C12E—H12J	109.1
N5A—C10A—C11A	112.22 (13)	C11E—C12E—H12I	109.1
H10A—C10A—H10B	107.9	C11E—C12E—H12J	109.1
C11A—C10A—H10A	109.2	H12I—C12E—H12J	107.8
			
O1A—N1A—C2A—C1A	35.4 (2)	O7D—N3D—C6D—C5D	179.93 (15)
O1A—N1A—C2A—C3A	−143.82 (17)	N1D—C2D—C3D—O3D	−0.7 (2)
O2A—N1A—C2A—C1A	−144.20 (17)	N1D—C2D—C3D—C4D	178.26 (14)
O2A—N1A—C2A—C3A	36.5 (2)	N2D—C4D—C5D—C6D	174.80 (15)
O3A—C3A—C4A—N2A	7.5 (2)	C1D—C2D—C3D—O3D	179.78 (16)
O3A—C3A—C4A—C5A	−172.60 (15)	C1D—C2D—C3D—C4D	−1.3 (2)
O4A—N2A—C4A—C3A	26.1 (2)	C2D—C1D—C6D—N3D	−176.99 (14)
O4A—N2A—C4A—C5A	−153.79 (16)	C2D—C1D—C6D—C5D	3.4 (2)
O5A—N2A—C4A—C3A	−154.60 (17)	C2D—C3D—C4D—N2D	−174.13 (14)
O5A—N2A—C4A—C5A	25.5 (2)	C2D—C3D—C4D—C5D	4.4 (2)
O6A—N3A—C6A—C1A	−179.68 (14)	C3D—C4D—C5D—C6D	−3.8 (2)
O6A—N3A—C6A—C5A	−1.3 (2)	C4D—C5D—C6D—N3D	179.98 (14)
O7A—N3A—C6A—C1A	0.9 (2)	C4D—C5D—C6D—C1D	−0.4 (2)
O7A—N3A—C6A—C5A	179.23 (15)	C6D—C1D—C2D—N1D	178.02 (14)
N1A—C2A—C3A—O3A	−4.7 (2)	C6D—C1D—C2D—C3D	−2.4 (3)
N1A—C2A—C3A—C4A	174.90 (14)	O1E—N1E—C2E—C1E	−33.6 (2)
N2A—C4A—C5A—C6A	174.32 (14)	O1E—N1E—C2E—C3E	145.62 (16)
C1A—C2A—C3A—O3A	176.05 (16)	O2E—N1E—C2E—C1E	143.49 (15)
C1A—C2A—C3A—C4A	−4.3 (2)	O2E—N1E—C2E—C3E	−37.3 (2)
C2A—C1A—C6A—N3A	−177.65 (14)	O3E—C3E—C4E—N2E	5.2 (2)
C2A—C1A—C6A—C5A	4.0 (2)	O3E—C3E—C4E—C5E	−171.92 (15)
C2A—C3A—C4A—N2A	−172.17 (14)	O4E—N2E—C4E—C3E	34.7 (2)
C2A—C3A—C4A—C5A	7.7 (2)	O4E—N2E—C4E—C5E	−147.92 (16)
C3A—C4A—C5A—C6A	−5.6 (2)	O5E—N2E—C4E—C3E	−144.93 (16)
C4A—C5A—C6A—N3A	−179.10 (14)	O5E—N2E—C4E—C5E	32.4 (2)
C4A—C5A—C6A—C1A	−0.8 (2)	O6E—N3E—C6E—C1E	−172.73 (14)
C6A—C1A—C2A—N1A	179.61 (14)	O6E—N3E—C6E—C5E	6.7 (2)
C6A—C1A—C2A—C3A	−1.2 (2)	O7E—N3E—C6E—C1E	8.1 (2)
O1B—N3B—C6B—C1B	−33.8 (2)	O7E—N3E—C6E—C5E	−172.40 (15)
O1B—N3B—C6B—C5B	145.57 (15)	N1E—C2E—C3E—O3E	−8.5 (2)
O2B—N3B—C6B—C1B	143.09 (15)	N1E—C2E—C3E—C4E	176.08 (13)
O2B—N3B—C6B—C5B	−37.5 (2)	N2E—C4E—C5E—C6E	−179.10 (14)
O3B—C5B—C6B—N3B	−9.6 (2)	C1E—C2E—C3E—O3E	170.70 (15)
O3B—C5B—C6B—C1B	169.72 (15)	C1E—C2E—C3E—C4E	−4.8 (2)
O4B—N2B—C4B—C3B	−150.42 (15)	C2E—C1E—C6E—N3E	177.47 (14)
O4B—N2B—C4B—C5B	29.8 (2)	C2E—C1E—C6E—C5E	−2.0 (2)
O5B—N2B—C4B—C3B	28.9 (2)	C2E—C3E—C4E—N2E	−179.32 (14)
O5B—N2B—C4B—C5B	−150.98 (15)	C2E—C3E—C4E—C5E	3.6 (2)
O6B—N1B—C2B—C1B	−175.70 (14)	C3E—C4E—C5E—C6E	−1.9 (2)
O6B—N1B—C2B—C3B	1.8 (2)	C4E—C5E—C6E—N3E	−178.58 (14)
O7B—N1B—C2B—C1B	4.4 (2)	C4E—C5E—C6E—C1E	0.9 (2)
O7B—N1B—C2B—C3B	−178.12 (14)	C6E—C1E—C2E—N1E	−176.60 (14)
N1B—C2B—C3B—C4B	−176.94 (14)	C6E—C1E—C2E—C3E	4.2 (2)
N2B—C4B—C5B—O3B	10.0 (2)	N4A—C7A—C9A—N5A	0.25 (19)
N2B—C4B—C5B—C6B	−174.48 (14)	N5A—C10A—C11A—C12A	75.73 (17)
C1B—C2B—C3B—C4B	0.5 (2)	C7A—N4A—C8A—N5A	0.13 (19)
C2B—C1B—C6B—N3B	−177.07 (13)	C8A—N4A—C7A—C9A	−0.24 (19)
C2B—C1B—C6B—C5B	3.6 (2)	C8A—N5A—C9A—C7A	−0.17 (18)
C2B—C3B—C4B—N2B	176.73 (14)	C8A—N5A—C10A—C11A	−99.79 (18)
C2B—C3B—C4B—C5B	−3.5 (2)	C9A—N5A—C8A—N4A	0.02 (19)
C3B—C4B—C5B—O3B	−169.83 (15)	C9A—N5A—C10A—C11A	81.85 (19)
C3B—C4B—C5B—C6B	5.7 (2)	C10A—N5A—C8A—N4A	−178.60 (14)
C4B—C5B—C6B—N3B	174.87 (13)	C10A—N5A—C9A—C7A	178.46 (15)
C4B—C5B—C6B—C1B	−5.8 (2)	C10A—C11A—C12A—N6A	−169.81 (13)
C6B—C1B—C2B—N1B	176.85 (13)	N4B—C7B—C9B—N5B	0.42 (19)
C6B—C1B—C2B—C3B	−0.6 (2)	N5B—C10B—C11B—C12B	77.05 (17)
O1C—N1C—C2C—C1C	−35.9 (2)	C7B—N4B—C8B—N5B	−0.2 (2)
O1C—N1C—C2C—C3C	145.17 (15)	C8B—N4B—C7B—C9B	−0.1 (2)
O2C—N1C—C2C—C1C	142.27 (16)	C8B—N5B—C9B—C7B	−0.53 (18)
O2C—N1C—C2C—C3C	−36.7 (2)	C8B—N5B—C10B—C11B	−96.84 (19)
O3C—C3C—C4C—N2C	−0.5 (2)	C9B—N5B—C8B—N4B	0.48 (19)
O3C—C3C—C4C—C5C	175.73 (15)	C9B—N5B—C10B—C11B	82.11 (19)
O4C—N2C—C4C—C3C	−36.2 (2)	C10B—N5B—C8B—N4B	179.59 (15)
O4C—N2C—C4C—C5C	147.29 (15)	C10B—N5B—C9B—C7B	−179.66 (14)
O5C—N2C—C4C—C3C	143.12 (16)	C10B—C11B—C12B—N6B	−167.69 (13)
O5C—N2C—C4C—C5C	−33.4 (2)	N4C—C7C—C8C—N5C	0.2 (2)
O6C—N3C—C6C—C1C	174.06 (14)	N5C—C10C—C11C—C12C	74.86 (18)
O6C—N3C—C6C—C5C	−5.9 (2)	C7C—N4C—C9C—N5C	0.14 (19)
O7C—N3C—C6C—C1C	−7.1 (2)	C8C—N5C—C9C—N4C	−0.01 (19)
O7C—N3C—C6C—C5C	172.89 (15)	C8C—N5C—C10C—C11C	79.6 (2)
N1C—C2C—C3C—O3C	0.4 (2)	C9C—N4C—C7C—C8C	−0.2 (2)
N1C—C2C—C3C—C4C	179.80 (13)	C9C—N5C—C8C—C7C	−0.12 (19)
N2C—C4C—C5C—C6C	179.99 (14)	C9C—N5C—C10C—C11C	−101.93 (19)
C1C—C2C—C3C—O3C	−178.42 (15)	C10C—N5C—C8C—C7C	178.57 (15)
C1C—C2C—C3C—C4C	1.0 (2)	C10C—N5C—C9C—N4C	−178.70 (15)
C2C—C1C—C6C—N3C	178.18 (14)	C10C—C11C—C12C—N6C	−166.96 (13)
C2C—C1C—C6C—C5C	−1.8 (2)	N5D—C7D—C9D—N4D	−0.43 (19)
C2C—C3C—C4C—N2C	−179.86 (14)	N5D—C10D—C11D—C12D	−70.02 (18)
C2C—C3C—C4C—C5C	−3.7 (2)	C7D—N5D—C8D—N4D	−0.51 (19)
C3C—C4C—C5C—C6C	3.7 (2)	C7D—N5D—C10D—C11D	−77.33 (19)
C4C—C5C—C6C—N3C	179.33 (14)	C8D—N4D—C9D—C7D	0.13 (19)
C4C—C5C—C6C—C1C	−0.7 (2)	C8D—N5D—C7D—C9D	0.56 (18)
C6C—C1C—C2C—N1C	−177.26 (14)	C8D—N5D—C10D—C11D	99.47 (18)
C6C—C1C—C2C—C3C	1.6 (2)	C9D—N4D—C8D—N5D	0.24 (19)
O1D—N1D—C2D—C1D	34.2 (2)	C10D—N5D—C7D—C9D	177.90 (15)
O1D—N1D—C2D—C3D	−145.42 (19)	C10D—N5D—C8D—N4D	−177.83 (14)
O2D—N1D—C2D—C1D	−142.63 (16)	C10D—C11D—C12D—N6D	162.89 (13)
O2D—N1D—C2D—C3D	37.8 (2)	N4E—C7E—C9E—N5E	−0.4 (2)
O3D—C3D—C4D—N2D	4.8 (2)	N5E—C10E—C11E—C12E	−73.02 (18)
O3D—C3D—C4D—C5D	−176.62 (16)	C7E—N4E—C8E—N5E	0.1 (2)
O4D—N2D—C4D—C3D	5.9 (3)	C8E—N4E—C7E—C9E	0.2 (2)
O4D—N2D—C4D—C5D	−172.77 (19)	C8E—N5E—C9E—C7E	0.41 (19)
O5D—N2D—C4D—C3D	−158 (2)	C8E—N5E—C10E—C11E	95.96 (19)
O5D—N2D—C4D—C5D	24 (2)	C9E—N5E—C8E—N4E	−0.3 (2)
O5DA—N2D—C4D—C3D	166.4 (11)	C9E—N5E—C10E—C11E	−80.7 (2)
O5DA—N2D—C4D—C5D	−12.3 (11)	C10E—N5E—C8E—N4E	−177.52 (15)
O6D—N3D—C6D—C1D	179.42 (15)	C10E—N5E—C9E—C7E	177.67 (15)
O6D—N3D—C6D—C5D	−0.9 (2)	C10E—C11E—C12E—N6E	165.79 (14)
O7D—N3D—C6D—C1D	0.3 (2)		

### Hydrogen-bond geometry (Å, º)

**Table:**

*D*—H···*A*	*D*—H	H···*A*	*D*···*A*	*D*—H···*A*
N6*A*—H6*AA*···O3*E*^i^	0.85 (2)	2.06 (2)	2.8943 (18)	167.5 (18)
N6*A*—H6*AB*···O3*D*^ii^	0.91 (2)	2.08 (2)	2.8671 (18)	143.1 (16)
N6*A*—H6*AB*···O4*D*^ii^	0.91 (2)	2.25 (2)	2.961 (2)	134.6 (15)
N6*A*—H6*AC*···N4*C*	0.95 (2)	1.87 (2)	2.8157 (19)	172.5 (18)
N6*B*—H6*BA*···O3*B*^iii^	0.92 (2)	2.117 (19)	2.8728 (17)	138.5 (15)
N6*B*—H6*BA*···O4*B*^iii^	0.92 (2)	2.268 (19)	3.006 (2)	136.6 (15)
N6*B*—H6*BB*···O3*B*	0.89 (2)	2.00 (2)	2.8502 (17)	161.0 (19)
N6*B*—H6*BC*···N4*A*^iv^	0.92 (2)	1.88 (2)	2.7988 (19)	173.4 (17)
N6*C*—H6*CA*···O2*C*^v^	0.85 (2)	2.305 (18)	2.8334 (19)	120.9 (15)
N6*C*—H6*CA*···O3*C*^v^	0.85 (2)	2.10 (2)	2.8944 (18)	155.4 (17)
N6*C*—H6*CB*···O3*A*^vi^	0.89 (2)	2.174 (19)	2.9145 (18)	140.2 (16)
N6*C*—H6*CB*···O4*A*^vi^	0.89 (2)	2.270 (19)	2.986 (2)	137.3 (15)
N6*C*—H6*CC*···N4*D*^vi^	0.92 (2)	1.91 (2)	2.8179 (19)	167.6 (17)
N6*D*—H6*DA*···O2*A*^vii^	0.89 (2)	2.361 (19)	2.9631 (19)	124.9 (15)
N6*D*—H6*DA*···O3*A*^vii^	0.89 (2)	2.06 (2)	2.8340 (17)	145.0 (16)
N6*D*—H6*DB*···O3*C*^vi^	0.89 (2)	2.16 (2)	2.9171 (17)	142.0 (17)
N6*D*—H6*DB*···O4*C*^vi^	0.89 (2)	2.27 (2)	2.9323 (19)	130.5 (16)
N6*D*—H6*DC*···N4*E*	0.89 (2)	1.91 (2)	2.7932 (19)	174.0 (17)
N6*E*—H6*EA*···O2*D*^viii^	0.91 (2)	2.32 (2)	2.973 (2)	128.7 (17)
N6*E*—H6*EA*···O3*D*^viii^	0.91 (2)	2.06 (2)	2.8527 (18)	145.4 (18)
N6*E*—H6*EB*···O3*E*	0.87 (2)	2.19 (2)	2.9056 (18)	139.6 (17)
N6*E*—H6*EB*···O4*E*	0.87 (2)	2.33 (2)	3.010 (2)	135.3 (16)
N6*E*—H6*EC*···N4*B*^viii^	0.93 (2)	1.85 (2)	2.7750 (19)	174.0 (19)
C8*A*—H8*A*···O2*B*^ix^	0.95	2.46	3.0887 (19)	123
C9*A*—H9*A*···O5*E*	0.95	2.43	3.243 (2)	144
C12*A*—H12*B*···O7*A*^ix^	0.99	2.46	3.313 (2)	145
C9*B*—H9*B*···O5*B*^i^	0.95	2.37	3.224 (2)	150
C12*B*—H12*D*···O7*C*^vi^	0.99	2.47	3.335 (2)	145
C8*C*—H8*C*···O5*C*^vi^	0.95	2.33	3.194 (2)	152
C9*C*—H9*C*···O2*E*^i^	0.95	2.49	3.105 (2)	123
C7*D*—H7*D*···O5*A*^vi^	0.95	2.35	3.233 (2)	155
C11*D*—H11*H*···O6*A*^vi^	0.99	2.55	3.421 (2)	147
C12*D*—H12*G*···O6*B*^vii^	0.99	2.43	3.238 (2)	139
C9*E*—H9*E*···O5*D*^ii^	0.95	2.27	3.162 (6)	156
C9*E*—H9*E*···O5*DA*^ii^	0.95	2.56	3.357 (17)	141
C12*E*—H12*I*···O6*E*^viii^	0.99	2.34	3.186 (2)	142

Symmetry codes: (i) *x*+1, *y*, *z*; (ii) *x*+1/2, −*y*+1/2, *z*+1/2; (iii) −*x*+1, −*y*+1, −*z*; (iv) −*x*+3/2, *y*+1/2, −*z*+1/2; (v) −*x*+2, −*y*+1, −*z*+1; (vi) −*x*+1, −*y*+1, −*z*+1; (vii) −*x*, −*y*+1, −*z*+1; (viii) *x*−1/2, −*y*+1/2, *z*+1/2; (ix) −*x*+3/2, *y*−1/2, −*z*+1/2.
